# Anti-*Anisakis* antibodies in colon cancer patients and their relationship with γδ T-cells

**DOI:** 10.1007/s00436-024-08216-y

**Published:** 2024-04-25

**Authors:** Juan C. Andreu-Ballester, Carmen Cuéllar, Javier Colmena-Zaragoza, Lorena Galindo-Regal, Carolina Hurtado-Marcos, Juan González-Fernández, Zutoia Balciscueta, Carlos García-Ballesteros, Francisca López-Chuliá, Ana I. Jiménez, Antonio Llombart-Cussac

**Affiliations:** 1 FISABIO Foundation–Public Health of Valencia, Spain and Parasitic Immunobiology and Immunomodulation Research Group (INMUNOPAR), Complutense University of Madrid, Madrid, Spain; 2 Departamento de Microbiología y Parasitología, Facultad de Farmacia, Universidad Complutense de Madrid, 28040 Madrid, Spain; 3 Emergency Medical Assistance Service (SAMU), Valencia, Spain; 4 Laboratory of Molecular Biology, Arnau de Vilanova Hospital, Valencia, Spain; 5 Laboratory of Parasitology and Immunology, San Pablo CEU University, Madrid, Spain; 6 Surgery Department, Arnau de Vilanova Hospital, Valencia, Spain; 7 Hematology Department, Arnau de Vilanova Hospital, Valencia, Spain; 8 Pathology Department, Arnau de Vilanova Hospital, Valencia, Spain; 9 Department of Medical Oncology in Arnau de Vilanova Hospital, Valencia, Spain

**Keywords:** *Anisakis*, Colon cancer, Specific antibodies, αβ T-cells, γδ T-cells, Apoptosis

## Abstract

Many pathogens are related to carcinogenesis. Chronic inflammation, as a result of persistent infection, leads to DNA damage, higher expression of oncogenes, decreased apoptosis and immunosuppression, which are some of the reasons for cancer induction. Among parasites, *Schistosoma*, *Opistorchis* and *Clonorchis* are recognised as infectious agents which contribute to cancer. A relationship between *Anisakis* and cancer was hypothesised because cellular responses to *Anisakis* products could result in inflammation and DNA damage. Previous research has shown a decrease in CD8+ γδ T-cells and an increase in αβ and γδ T-cell apoptosis in colon cancer (CC) samples. Ninety-two CC patients and 60 healthy subjects were recruited. γδ and αβ T-cells were analysed, and their apoptosis was evaluated. Anti-*Anisakis* antibodies were tested in sera from CC patients and controls. Anti-*Anisakis* IgG, IgM, IgA and IgE antibodies were significantly higher in CC patients. A significant increase in anti-*Anisakis* IgA levels was observed in patients with angiolymphatic invasion. The number of all γδ T-cells, as well as CD3+ CD4+ αβ T-cells, was significantly lower in CC patients. The apoptosis of all T-cells was significantly increased in patients with CC. We observed a significantly higher percentage of anti-*Anisakis* IgE positive patients having a deficit of CD3+ γδ T-cells. Our results suggest a relationship between *Anisakis* and CC.

## Introduction

In 2019, with almost 24 million cases and nearly 10 million deaths, cancer was the second leading cause of death (after cardiovascular disease) worldwide. Specifically, colon cancer (CC) ranks second in annual incidence after tracheal, bronchus and lung cancers, with more than two million cases and at least one million deaths per year. According to GLOBOCAN 2020, the global cancer burden will increase and is expected to be 28.4 million cases by 2040 (Global Burden of Disease Cancer 2022; Sung et al. 2021). Many pathogens, such as bacteria, viruses, fungi and parasites, are associated with carcinogenesis in 20% of cancers. Chronic inflammation, as a result of persistent infection, leads to DNA damage, higher expression of oncogenes, decreased apoptosis and immunosuppression, which are some of the reasons for cancer induction. Immunosuppression leads to carcinogenesis via immunological disruption by inhibiting T-cell responses to mitogens and antigens (Hatta et al. 2021). Specifically, CC were linked to bacteria, such as *Bacteroides fragilis*, *Streptococcus gallolyticus*, *Streptococcus bovis* and *Fusobacterium nucleatum* (Shang and Liu 2018). Among parasites, *Schistosoma haematobium* (bladder cancer), *Opistorchis viverrini* and *Clonorchis sinensis* (cholangiocarcinoma) are recognised by the International Agency for Research on Cancer as major infectious agents contributing to the global number of cancers*.* Similarly, *Schistosoma japonicum* has been implicated in several human malignancies, such as liver and colorectal cancers (Hamid 2019; Jain et al. 2019; Hatta et al. 2021; Jain and Rana 2023). *Cryptosporidium* has also been associated with colon cancer (Sawant et al. 2020). Recently, our group described a high prevalence of microsporidia in tissues of patients with CC, also observing an increase in specific IgG and IgE in the serum of these patients (Redondo et al. 2022). *Anisakis* is a nematode that parasitises the stomach of marine mammals. Eggs produced by females are shed into water where they embryonate. Marine crustaceans ingest free-living larvae, and fish become infected when they eat infected crustaceans. The cycle is completed when infected fish are eaten by marine mammals. Humans can act as accidental hosts by consuming raw or undercooked fish. The larvae can survive for up to weeks. Parasites located in the gastric or intestinal mucosa may cause gastrointestinal infection. *Anisakis* can also cause IgE-mediated reactions such as urticaria, angioedema, asthma and anaphylaxis. This food-borne parasite causes serious public health problem worldwide (Morozinska-Gogol 2019). Almost three decades ago, the *A. simplex* parasite was suggested to be a co-factor of gastric cancer (Petithory et al. 1990). Specific antibodies against *A. simplex* were found in patients with gastrointestinal cancer, especially with a higher rate of positivity for anti-*Anisakis* IgA (Gutierrez and Cuellar 2002). Subsequently, the *Anisakis* larval products were able to induce inflammation and cell proliferation (or inhibition of apoptosis), demonstrating a relationship between the parasite and cancer. Similarly, *Anisakis* can cause DNA damage by elevating the induction of host p53 (Messina et al. 2016). Moreover, *Anisakis* crude extract induced strong inflammatory responses in the intestinal epithelium (Speciale et al. 2017). Furthermore, it was proposed that *Anisakis* could have tumourigenic potential because larval products increased cell proliferation, decreased apoptosis and induced changes in the expression of serum cancer-related miRNAs in epithelial cells (Corcuera et al. 2018).

γδ T-cells represent 5–10% of T lymphocytes in the peripheral blood, but reach up to 50% of mucosal intra-epithelial T lymphocytes. With characteristics between innate and adaptive immunity, they participate in the first line of defence against pathogens and tumoural cells, currently being a source of study for the application of treatments in some types of tumours (Ma et al. 2020; Poggi and Zocchi 2014; Silva-Santos et al. 2019). Recently, we described a decrease in cytotoxic CD8+ γδ T-cells and an increase in apoptosis in all αβ and γδ T-cell subsets in patients with CC *vs* healthy subjects. In addition, γδ T-cells decreased in the peripheral blood of patients with microscopic infiltration in tissues, a history of cancer and synchronous colon cancer. These findings suggest a crucial role for γδ T-cells in cancer immunomodulation (Andreu-Ballester et al. 2022).

The main objective of this study was to analyse the levels of anti-*Anisakis* antibodies in the serum of newly diagnosed cancer patients without any specific immunosuppressive treatment and to relate them to T-cell immunity and other clinical parameters of colon cancer.

## Materials and methods

### Ethics statement

The Research Ethics Committee of Arnau de Vilanova Hospital, Valencia (Spain) approved this study, and each patient signed an informed consent document. This study was conducted following the recommendations of the Spanish Bioethics Committee, Spanish legislation on Biomedical Research (Law 14/2007, July 3) and Personal Data Protection (Spanish Law 3/2018 and European Law UE676/2018) in accordance with The Code of Ethics of the World Medical Association (Declaration of Helsinki). Pseudonymity of the participants was ensured.

### Subjects of study and clinical samples

In this prospective study of cases and controls, 92 patients with histopathologically confirmed colon cancer were recruited and diagnosed in our hospital in the last year. Patients with rectum cancer were excluded from this study. Sixty healthy subjects without cancer (control) were included in this study based on the following criteria: absence of inflammatory, autoimmune, acute infections or known immunodeficiency diseases; and no immunosuppressive or antibiotic treatment or any kind of vaccine during the previous 6 months. Patients with cancer should meet the same criteria. In addition, they should not have received any specific treatment for their disease, chemotherapy, radiotherapy or immunological drugs.

### Cell isolation for analysis of γδ and αβ T-cells and apoptosis evaluation

Peripheral blood was collected in tubes containing EDTA, and mononuclear cells (MNCs) were obtained by centrifugation on a density gradient (Lymphoprep™ (Palex Medical SA)). Apoptosis level was analysed by using the ANNEXIN V-FITC/7-AAD kit according to the manufacturer instructions (Beckman Coulter Life Sciences, Miami, USA).

To analyse γδ and αβ T-cell subsets, 100 μl of MNCs was incubated with 10 μl of the following monoclonal antibodies (all from Beckman Coulter, Inc., Miami, USA): γδ T-cells, anti-TCR PAN γδ-PE, anti-TCR PAN γδ-PE, CD56-PC7, CD4-A750-APC, CD3-A700-APC, CD8-PB, and CD45-KRO, and αβ T-cells, anti-TCR PAN αβ-PE, CD56-PC7, CD4-A750-APC, CD3-A700-APC, CD8-PB and CD45-KRO.

A total of 100.000 cells were acquired using a multiparameter Navios flow cytometer, and data were analysed using Kaluza software (both from Beckman Coulter Life Sciences, Miami, USA). “Kaluza Analysis Flow Cytometry Software is designed to simply and efficiently analyse multicolour data. Four persistent control panels provide access to every aspect of the data. Any changes made are automatically reflected in the analysis. The statistics are updated immediately, and the population colours change, giving the operator real-time feedback”. (https://www.mybeckman.co/resources/product-applications/cell-based-therapeutics/automated-cellular-analysis/kaluza-analysis-software).

### *Anisakis* antigen and determination of specific antibodies

*Anisakis* L3 were extracted from blue whiting (*Micromesistius poutassou*) and homogenised after their sonication and extraction in PBS as previously described (Garcia-Palacios et al. 1996).

ELISA plates (Costar, Corning, NY, USA) were sensitised by the addition of 10 μg/ml total larval antigen. Human sera were added at 1/100 in PBS-Tween with 0.1% BSA and incubated. Peroxidase (HRP)-conjugated goat anti-human immunoglobulins (Ig’s, IgM, IgG or IgA; Biosource International, Camarillo, CA, USA) were used (Daschner et al. 2002; Gutierrez and Cuellar 2002). For IgE determination, sera were added at 1/2 dilution. Subsequently, a murine anti-human IgE monoclonal antibody (IgG1Ƙ, E21A11, INGENASA, Madrid, Spain) was added followed by incubation with goat anti-murine IgG1 antibodies conjugated with HRP (Life Technologies, Grand Island, NY, USA). Anti-*Anisakis* antibody positivity was determined using the following formula: mean optical density + one standard deviation in healthy subjects.

### Statistical analyses

Student’s *t test* was used to compare the quantitative variables if normality was assumed (Kolmogorov-Smirnov test). When normality was not accepted, the Mann-Whitney *U* test was used. To compare categorical variables, the OR (IC95%) in the Fisher’s exact test was used. Correlation studies using Spearman’s Correlation Coefficient were performed to compare αβ and γδ T-cell apoptosis with the anti-*Anisakis* antibody levels. Statistical significance was set at *p* < 0.05. To assess T-cell deficiency, values below the 25th percentile were considered. Data were analysed using the Statistical Package for Social Sciences (SPSS 19.0; SPSS Inc., Chicago, IL, USA). Figures were generated using GraphPad Prism version 8.0.0 (GraphPad Software, San Diego, CA, USA).

## Results

The characteristics of the patients with colon cancer are summarised in Table 1.

**Table 1:** Characteristics of patients with colon cancer (*n* = 92)

	Mean ± SD		*N* (%)
Age	69.2±11.6	Histologic type (SNOMED)	
CEA (ng/ml)	8.5 ± 17.9	- Adenocarcinoma (AC)	73 (79.3)
CA 19-9 antigen (µ/ml)	15.6 ± 29.6	- Mucinous AC	12 (7.9)
Gender	*N* (%)	- Infiltrant on Adenoma AC	6 (6.5)
- Male	59 (62.1)	- Neuroendocrine AC	1 (1.1)
- Female	33 (57.9)	Grade	
TNM staging		-Low grade	49 (53.3)
- I	31 (33.7)	-High grade	43 (46.7)
- II	26 (28.3)	Invasion	
- III	31 (33.7)	- Subserosa	42 (45.7)
- IV	4 (4.4)	- Muscular	28 (30.4)
Polyposis	55 (59.8)	- Submucosa	12 (13.0)
Recurrent	7 (7.6)	- Neighbouring organ	5 (5.4)
Synchronous cancer	7 (7.6)	- Serosa visceral	4 (4.3)
Tissue	Mean ± SD	- *In situ*	1 (1.1)
Analysed lymph nodes	17.1 ± 7.8	Angiolymphatic invasion	34 (36.9)
Lymph nodes metastasis	1.2 ± 2.2	Perineural invasion	4 (4.3)

*SD* standard deviation, histologic type (OMS) SNOMED (Systematized Nomenclature of Medicine) code, *TNM* tumour-Node-Metastasis, *CEA* carcinoembryonic antigen, *CA* carbohydrate antigen

### Evaluation of anti-*Anisakis* antibody levels in serum of colon cancer (CC) patients vs controls

**Fig. 1:**
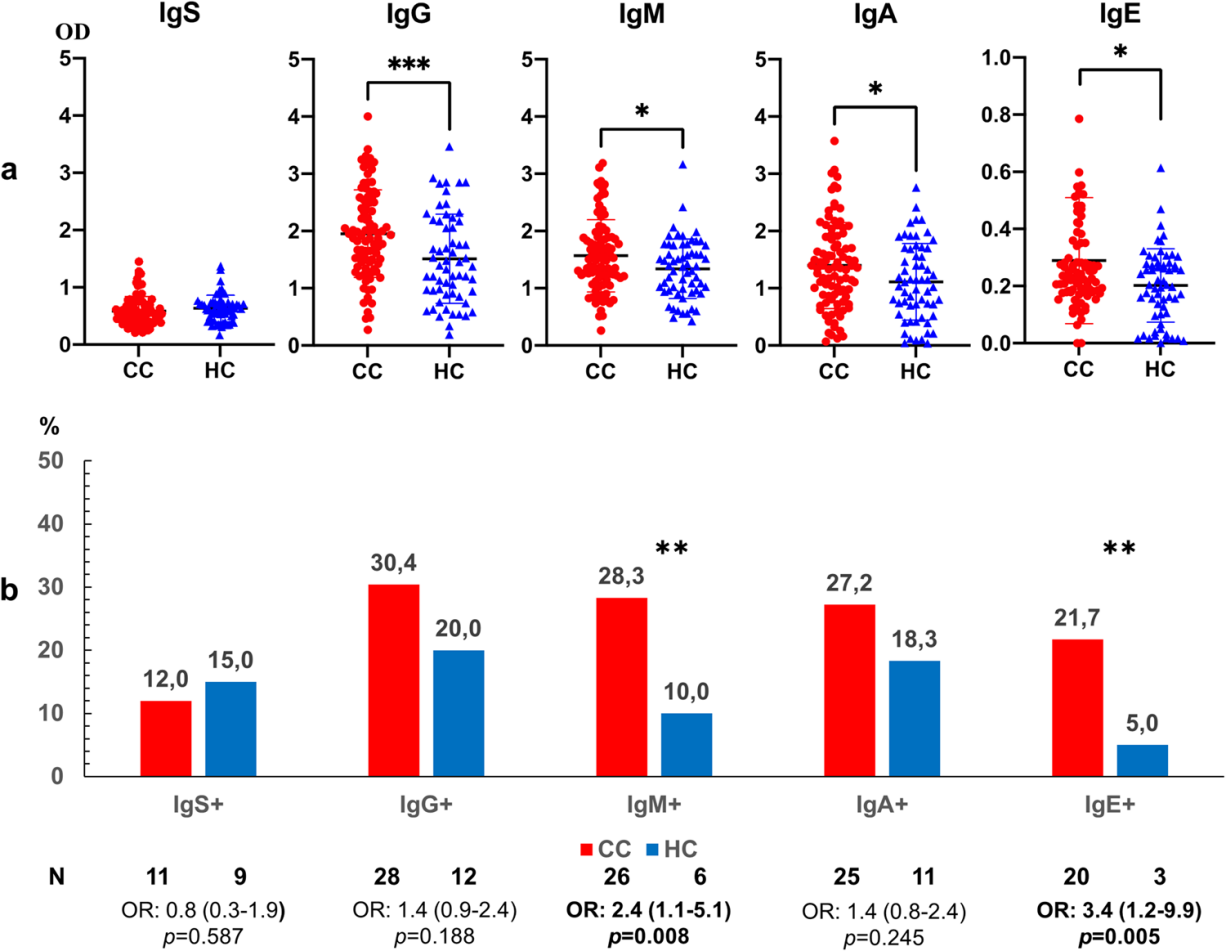
Panel **a**. Anti-*Anisakis* immunoglobulins in serum of patients with colon cancer (CC, *n* = 92) *vs* healthy control subjects (HC, *n* = 60). Values are expressed as the mean optical density (OD). T-bars denote the standard deviation. The Mann-Whitney *U* test was used. (***p < 0.001 and *p < 0.05). Panel **b**. Percentage of positive sera (mean OD + 1 standard deviation) in CC patients and healthy subjects. Odds ratio, OR: (IC95%), Fisher’s exact test was used. (** *p* < 0.01)

Anti-*Anisakis* IgG, IgM, IgA and IgE levels were significantly higher in serum from CC patients as compared to healthy control subjects (Fig. 1a). The percentages of positive sera were significantly higher in CC patients for both IgM and IgE anti-*Anisakis* specific antibodies (Fig. 1b). Logistic regression analysis showed that the results had even more statistical power. Positive anti-*Anisakis* IgM: Step 1, Exp B = 3.5 (CI95%, 1.3–9.4) and positive anti-*Anisakis* IgE: Exp B = 5.3 (CI95%, 1.5–18.9), *p* < 0.001.

### Evaluation of anti-*Anisakis* antibodies in serum of colon cancer (CC) patients according to differentiation grade

**Fig. 2:**
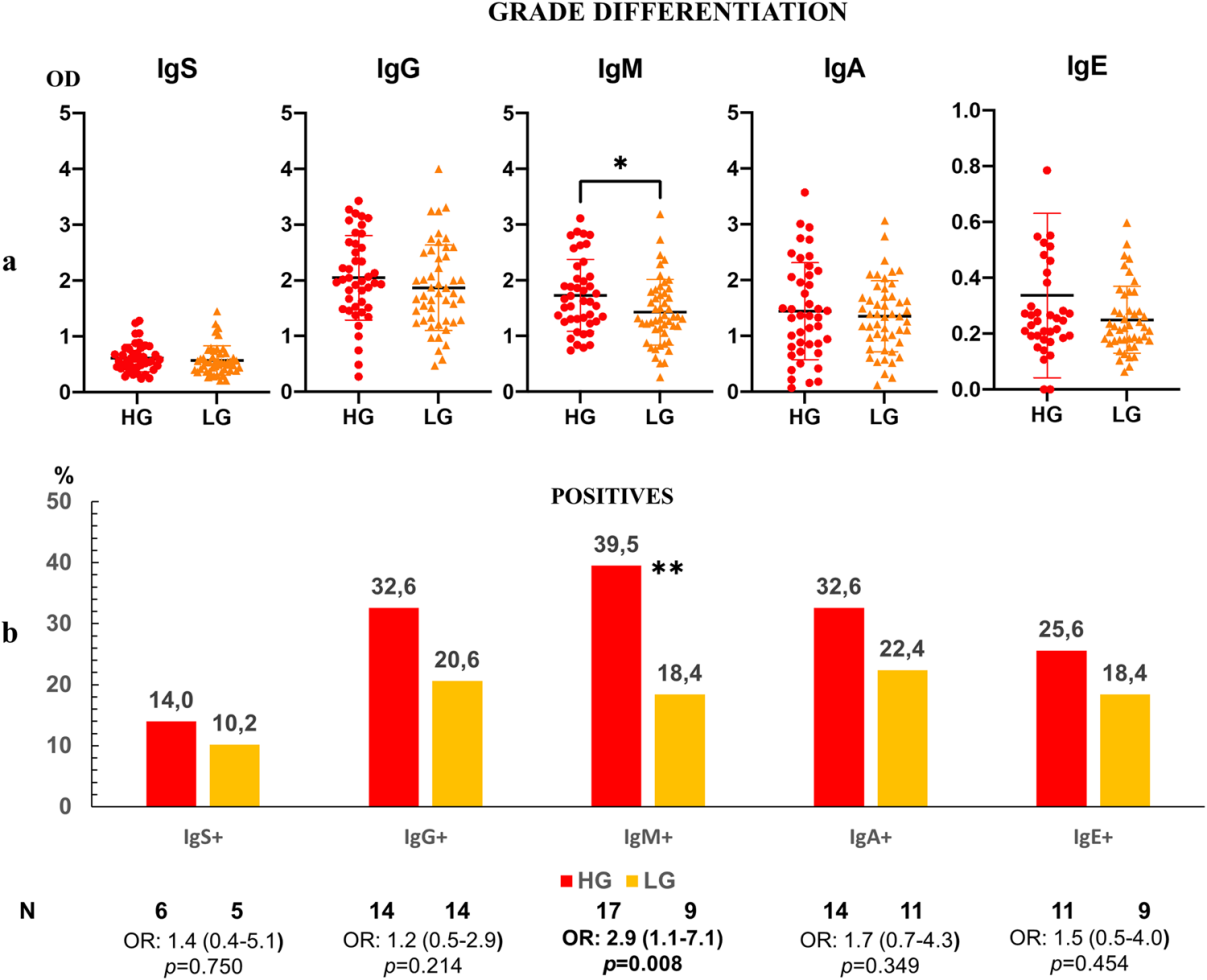
Panel **a**. Anti-*Anisakis* immunoglobulins in the serum of patients with colon cancer (CC, *n* = 92) according to high-grade (HG, *n* = 43) and low-grade (LG, *n* = 49) of tumour differentiation grade. Values are expressed as mean optical density (OD). T-bars denote standard deviation. The Mann-Whitney *U* test was used. (*p < 0.05). Panel **b**. Percentage of positive sera (mean OD + 1 standard deviation) in the high-grade and low-grade groups. Odds ratio, OR: (IC95%), Fisher’s exact test was used. (**p < 0.01)

The levels of anti-*Anisakis* immunoglobulins were higher in CC patients with high-grade dysplasia in their tumour cells. IgM values were statistically different between the groups according to differentiation grade (Fig. 2a). The percentages of positive sera were also higher in patients with a high differentiation grade in their tumour cells, which was significant for patients with positive anti-*Anisakis* IgM (Fig. 2b).

### Evaluation of anti-*Anisakis* antibodies in serum of colon cancer (CC) patients according to the presence of additional polyps in colon

**Fig. 3:**
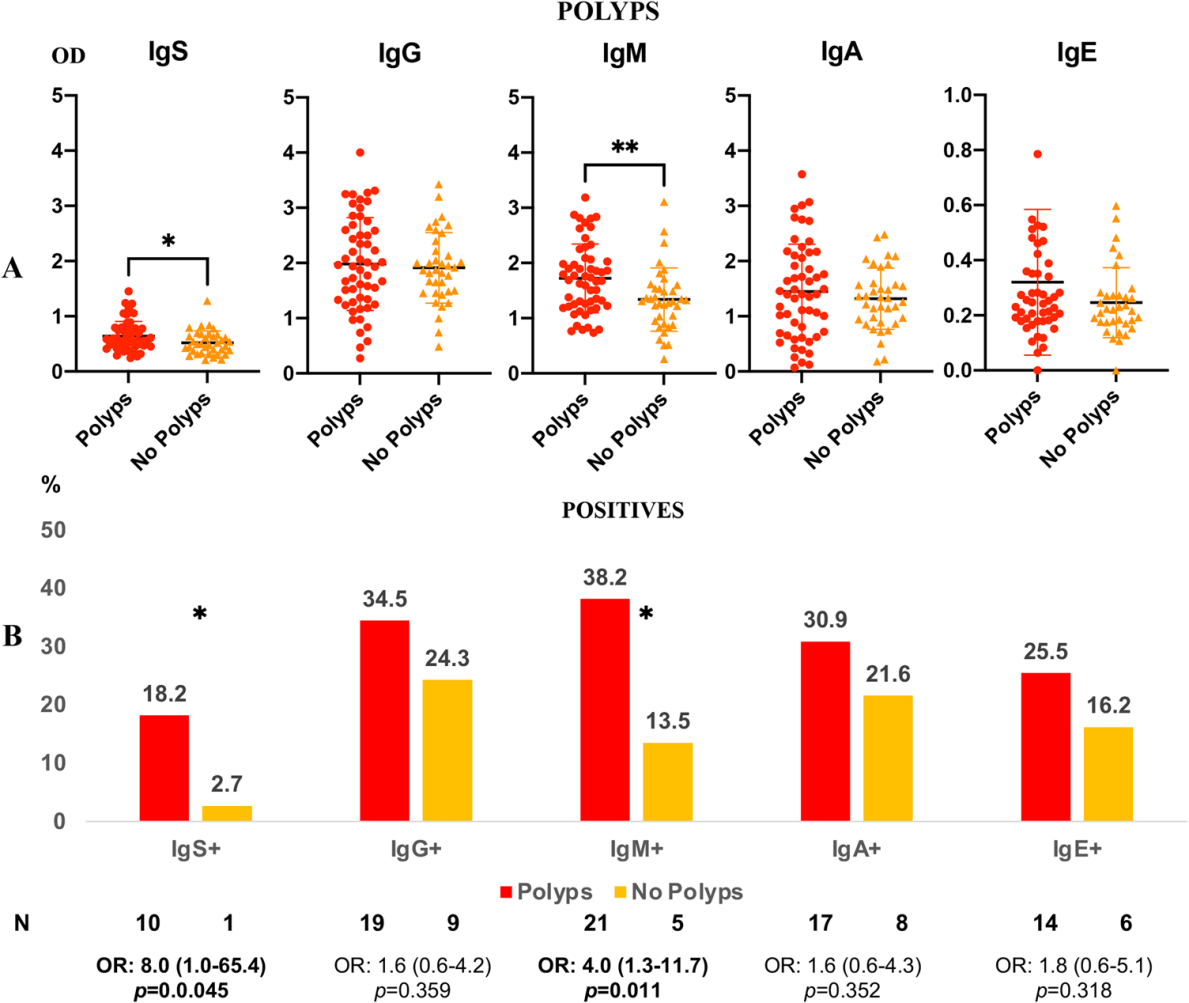
Panel **A**. Anti-*Anisakis* immunoglobulins in the serum of patients with colon cancer (CC, *n* = 92) according to polyposis (*n* = 55) or non-polyposis (*n* = 37) in the colon. Values are expressed as the mean optical density (OD). T-bars denote the standard deviation. The Mann-Whitney *U* test was used. (**p < 0.01 and *p < 0.05). Panel **B**. Percentage of positive sera (mean OD + 1 standard deviation) in polyposis or non-polyposis. Odds ratio, OR: (IC95%), Fisher’s exact test was used. (*p < 0.05)

We analysed anti-*Anisakis* immunoglobulins in CC patients with polyps in other locations of the colon in addition to the tumour areas. These results were compared to those obtained from patients with CC without polyps. Anti-*Anisakis* Ig’s and IgM antibodies were significantly higher in patients with additional polyps than in those without polyps (Fig. 3a). In addition, in the group of patients with polyps, the percentages of anti-*Anisakis* Ig’s and IgM positive sera were significantly higher than those in patients without polyps. The percentages of anti-*Anisakis* IgG, IgA and IgE positive sera were also higher in patients with polyps, although the difference was not statistically significant (Fig. 3b).

### Evaluation of anti-*Anisakis* antibodies in serum of colon cancer (CC) patients according to the presence of angiolymphatic invasion (AI)

**Fig. 4:**
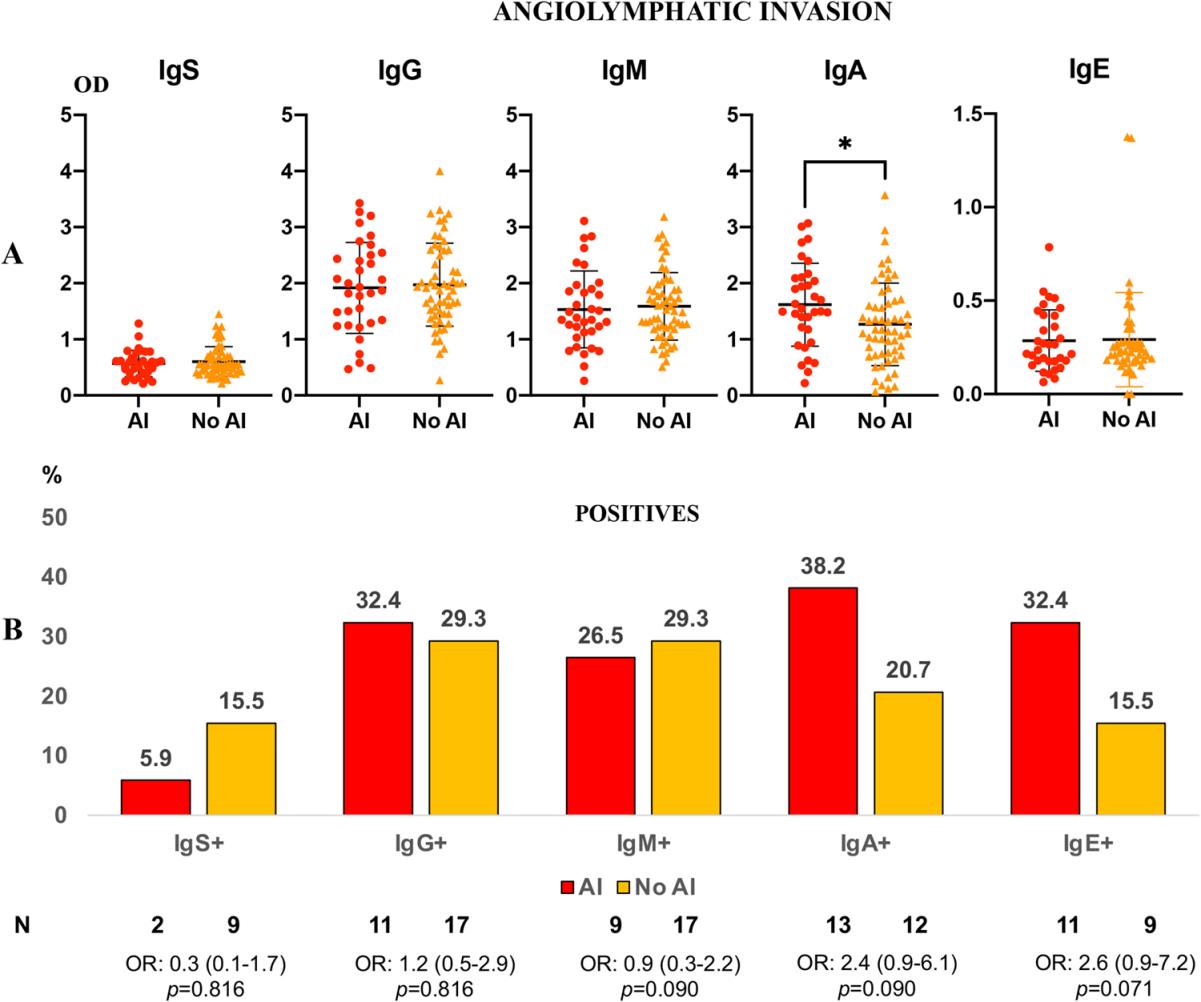
Panel **a**. Anti-*Anisakis* immunoglobulins in the serum of patients with colon cancer (CC, *n* = 92) according to angiolymphatic invasion (AI, *n* = 34) or not (*n* = 58). Values are expressed as means of optical density (OD). T-bars denote standard deviation. Mann-Whitney *U* test was used. (*p < 0.05). Panel **b**. Percentage of positive sera (mean OD + 1 standard deviation) in angiolymphatic invasion. Odds ratio, OR: (IC95%), Fisher’s exact test was used

A significant increase in anti-*Anisakis* IgA levels was observed in CC patients with AI (Fig. 4a). In addition, there was an increase in the anti-*Anisakis* IgA (*p* = 0.09) and IgE (*p* = 0.071) percentages of positive patients in the CC group with AI *vs* non-AI (Fig. 4b).

### Differences in counts and apoptosis of αβ and γδ T-cells according to the presence of colon cancer (CC)

**Fig. 5:**
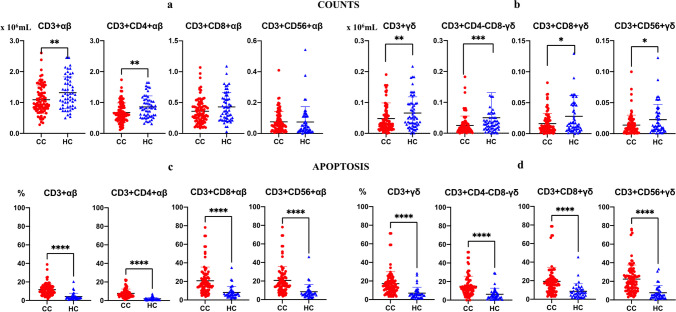
Differences in apoptosis of αβ (panels **a** and **c**) and γδ (panels **b** and **d**) T-cells according to colon cancer (Red/CC) (*n* = 92) and healthy control subjects (Blue/HC) (*n* = 60). Values are expressed as mean (×106/ml). T-bars denote standard deviation. Mann-Whitney *U* test was used. (****p < 0.0001, ***p < 0.001, **p < 0.01 and *p < 0.05)

The number of all γδ T-cell subsets was significantly lower in patients with CC (Fig. 5b). A significant reduction in the CD3+CD4+ αβ T-cell subset was also observed in patients with CC (Fig. 5a). Apoptosis of all αβ and γδ T-cell subsets was significantly increased compared with that in healthy control subjects (Fig. 5c, d).

### Correlation of anti-*Anisakis* antibodies and T-cell subsets

**Fig. 6:**
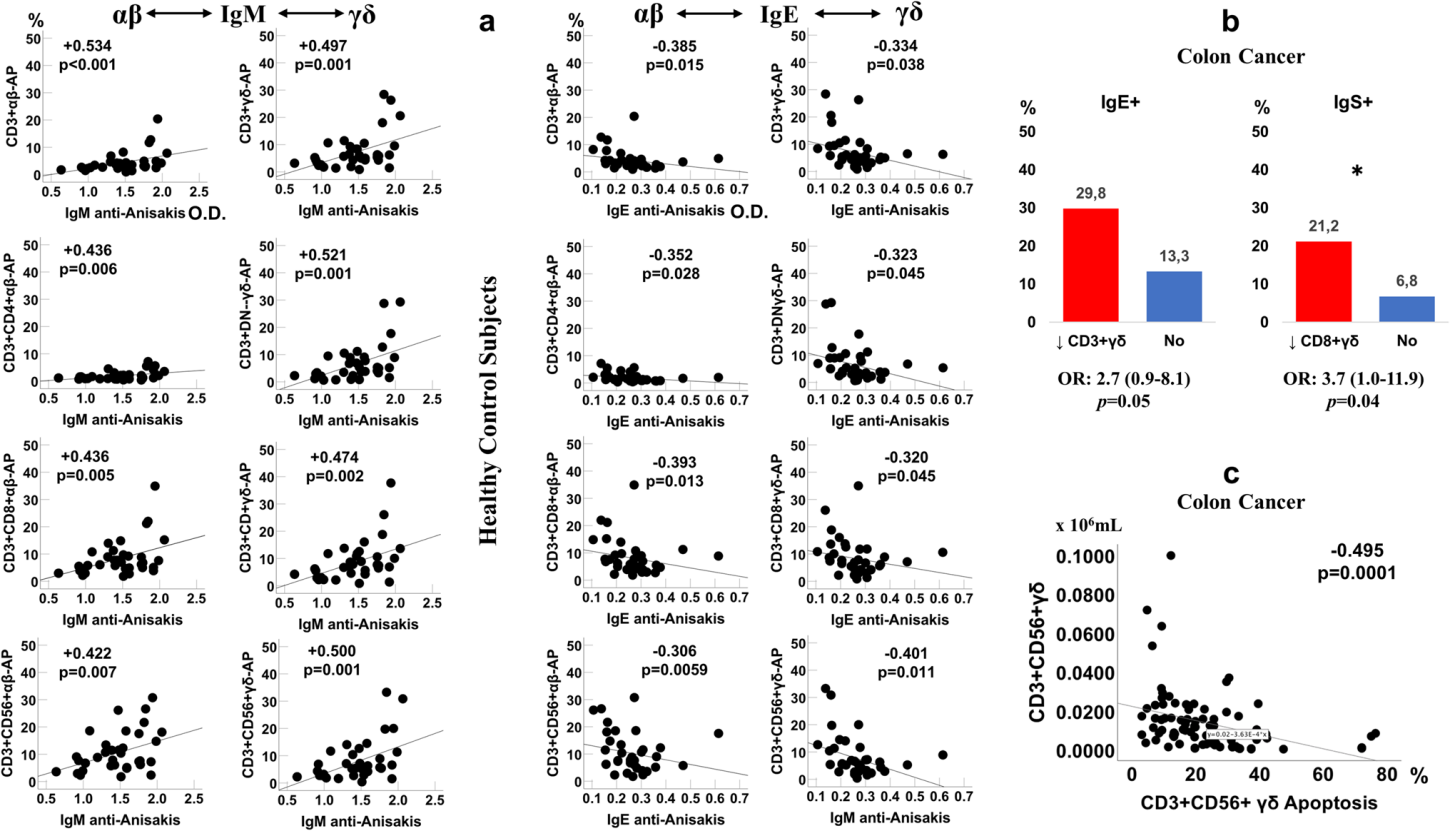
Correlations between αβ and γδ cell apoptosis (AP) (%) and anti-*Anisakis* IgM and IgE antibodies (optical density: OD), *n* = 40 (Panel **a**). Percentage of anti-*Anisakis* IgE and Ig’s positive sera in colon cancer patients with or without CD3+ and CD8+ γδ T-cell deficiency, Odds ratio, OR: (IC95%), and Fisher’s exact test were used (Panel **b**). Correlation between apoptosis and number of CD3+ CD56+ γδ T-cells in CC patients (Panel **c**). Spearman’s rank correlation coefficient was used

A correlational study was conducted to assess the relationship between anti-*Anisakis* antibody levels and the αβ and γδ T-cell subsets. We found a direct relationship between all T-cell subsets and anti-*Anisakis* IgM in healthy subjects. Conversely, a negative correlation between all T-cell subsets and anti-*Anisakis* IgE was observed in the control group (Fig. 6a). However, no correlation was observed in patients with CC.

Later, we investigated the relationship between anti-*Anisakis* positivity and deficits in αβ and γδ T-cell populations in patients with CC. We observed a significantly higher percentage of positive anti-*Anisakis* IgE and Ig’s sera in the group of patients having a deficit of CD3+ γδ T and CD3+ CD8+ γδ T-cells, respectively (Fig. 6b).

As a result of the study, we found a negative correlation between CD3+ CD56+ γδ T-cells and apoptosis in patients with CC (Spearman test: −0.495, *p* < 0.0001) (Fig. 6c).

## Discussion

To our knowledge, this is the first study to investigate the levels of all anti-*Anisakis* immunoglobulin isotypes in CC patients, relating them to αβ and γδ T-cell immunity. The first outstanding finding of the investigation was the significant increase in the levels of all anti-*Anisakis* specific isotypes in CC patients *vs* control subjects, with significantly higher IgM and IgE positivity rates in CC patients. In a previous study, we demonstrated an increase in anti-*Anisakis* antibodies in digestive pathologies, and particularly in specific IgA in gastrointestinal cancer (Gutierrez and Cuellar 2002). Our results could be explained by the deficit of T-cells in patients with CC described in the present study, especially γδ T-cell subsets. We also previously demonstrated that anti-*Anisakis* antibodies increased in sepsis, a pathology evolving from immunosuppression, particularly related to γδ T-cells. Additionally, IgG, IgA and IgE were related to the highest sepsis severity stages with higher immunosuppression (Andreu-Ballester et al. 2018). In a previous study, we observed that apoptosis was significantly increased in all conventional T lymphocytes and both αβ and γδ T-cell subsets in patients with CC compared to healthy subjects (Andreu-Ballester et al. 2020). Interestingly, in a study conducted in septic patients, apoptosis increased progressively according to the severity of the septic process, especially in non-surviving patients. This phenomenon was especially significant in CD3+CD56+ γδ T-cells (Andreu-Ballester et al. 2022). It seems that there is a similarity in the immune behaviour between infections and cancer: decreased T-cell immunity and increased apoptosis. Furthermore, this relationship is more intense in the case of γδ T-cells. This raises the question of whether a pathogen that maintains a persistent inflammatory response, and therefore an immune reaction similar to that presented in sepsis, is involved in cancer.

Traditionally, the histological grade of colorectal carcinoma is assessed based on the percentage of glandular differentiation in the tumour. Well-differentiated adenocarcinoma or grade 1 shows > 95% gland formation; moderately differentiated or grade 2 shows 50–95% gland formation and poorly differentiated or grade 3 is mostly solid with < 50% gland formation. Grade 1 was considered low grade, and grades 2 and 3 were high grade. High-grade tumours tend to grow and spread more rapidly to other tissue and therefore have a poor prognosis (Fleming et al. 2012; Hamilton et al. 2010). The highest antibody levels and the highest positivity rates for anti-*Anisakis* IgM were found in patients with high-grade CC, while the lowest levels were observed in low-grade CC. This could indicate increased risk of *Anisakis* infection in patients had a poor prognosis (high grade), although significant differences were not observed in the αβ and γδ T-cell subsets between high grade and low grade (data not shown).

Although it may take up to 10 years for some polyps to become cancerous, the polyposis-adenoma-carcinoma sequence is the form demonstrated by a growing body of scientific evidence, in which only a small percentage of polyps acquire malignant features (Oines et al. 2017). Although most of polyps will never develop into colorectal cancer, multiple somatic mutations in the epithelial cell lineages, already pre-existing in the polyp-adenoma stage, effectively contribute to the development and progression of malignancy (Sullivan et al. 2022). In this study, we analysed the presence of additional polyps to the tumour lesions found during colonoscopy and in the tissue sample. We also demonstrated a significant increase in anti-*Anisakis* IgM levels and the percentage of subjects with positive outcome in the group of CC patients with additional polyps. This suggests a clear relationship between *Anisakis* and the presence of polyps because there was an increase in all anti-*Anisakis* immunoglobulins in patients with additional polyps.

Angiolymphatic invasion (AI) is considered a strong risk factor correlated with adverse outcome. The prognostic significance of submucosal small vessel involvement is associated with an increased risk of regional lymph node metastasis, and the involvement of extramural venous invasion is associated with an increased risk of liver metastasis (Compton et al. 2000). IgA is the most abundant antibody isotype in mucosal secretions but is also present at significant concentrations in serum (de Sousa-Pereira and Woof 2019). IgA clearly plays a critical role in protection of mucosal surfaces against attack by invading pathogens and presumably is able to kill malignant cells at mucosal sites. Gutiérrez and Cuéllar found high levels of anti-*Anisakis* IgA in patients with digestive pathologies, including gastrointestinal cancer (Gutierrez and Cuellar 2002). In our study, we confirmed an increase in anti-*Anisakis* IgA in patients with CC and AI. If there is an increase in *Anisakis* infection in a situation of decreased T-cell immunity and with AI, it seems logical that the immune system mounts a response with an increase in the most abundant antibody isotype associated with mucous membranes, resulting in further increases in the serum levels of IgA antibodies specific for *Anisakis*.

The allergic IgE-mediated immune reaction in the course of *Anisakis* infection involves a parallel secondary Th2 type memory response and a primary immunological stimulation of both Th2 and Th1 lymphocyte subsets (Daschner et al. 2002). The anti-inflammatory and pro-tumoural role of the Th2 cellular immune response is well established. Differentiation of Th2 cells depends on IL-4 secreted by B cells, natural killer T-cells (NKT), CD4+ *naïve* T-cells and mast cells, via Signal Transducer and Activator of Transcription 6 (STAT6) signalling (Basu et al. 2021; Maier et al. 2012; Nappo et al. 2017). STAT6 overexpression, detected in the immune microenvironment of cancer, promotes immunosuppression and metastasis formation by promoting angiogenesis (Fu et al. 2019).

Anti-*Anisakis* antibodies are present in all isotypes, reflecting a broad immune response involving both Th2 and Th1 lymphocyte subtypes. Specific IgG, IgA and IgE reached the highest values by 1 month after exposure to larval antigens. Specific IgM levels showed the highest values early in the infection and decrease continuously over the next 6 months (Daschner et al. 2002). Our results suggest the possibility of a remote *Anisakis* infection overt time due to the elevated production of IgG and IgE with reinfections with the parasite close to the CC diagnosis. This was demonstrated by the high serum of IgM and IgA levels in high-grade tumours and those progressing with AI, respectively. There are experimental studies suggesting relationships between *Anisakis* and the alteration of epithelial cells and their possible malignant transformation (Bellini et al. 2022; Speciale et al. 2017). Similarly, our results clearly link *Anisakis* to CC. However, further research is needed to demonstrate whether this parasitism is a tumour-producing factor or a consequence of T-cell deficiency independent of the cancer aetiology. In addition, immunodeficiency could favour the presence of the parasite which, in turn, would alter apoptosis, leading to possible oncogene activation with malignant transformation of cells.

In conclusion, all the different isotypes of anti-*Anisakis* antibodies were significantly higher in the colon cancer patient group than in controls. The percentages of anti-*Anisakis* IgM and IgE positive sera were also significantly higher in CC patients compared to healthy controls. The tumour differentiation grade and polyposis were related to high levels and positivity rates of anti-*Anisakis* IgM*.* The highest serum anti-*Anisakis* IgA levels were found in patients who were affected by angiolymphatic invasion. The frequencies in the γδ T-cell compartment and CD4+ αβ T-cells were significantly lower in patients with CC compared to the control group. Apoptosis was significantly increased in both αβ and γδ T-cell subsets in patients with CC compared to healthy control subjects. In healthy subjects, there was a direct relationship between all T-cell subsets and anti-*Anisakis* IgM and an indirect relationship between all T-cell subsets and anti-*Anisakis* IgE. These relationships were not observed in CC patients. The results described above suggest an association between *Anisakis* and colon cancer. However, based on the obtained results, we can only discuss an association between cancer and *Anisakis*. To establish a potential cause-and-effect relationship, we would require experiments, such as those conducted in animal models, to clearly demonstrate the induction of colon cancer following infection with this parasite. Likewise, to assess whether *Anisakis* affects the numbers, apoptosis and differentiation of T cells in tumours, it is necessary to expose T lymphocytes extracted from tumour tissues to *Anisakis* antigens and examine their differentiation phases, while also assessing cell proliferation and apoptosis.

## Data Availability

The data that support the findings of this study are available from the corresponding author upon reasonable request.

## References

[CR1] Andreu-Ballester JC (2018). Anti-Anisakis sp. antibodies in serum of patients with sepsis and their relationship with gammadelta T cells and disease severity. Int J Parasitol.

[CR2] Andreu-Ballester JC (2020). Differences in circulating gammadelta T cells in patients with primary colon cancer and relation with prognostic factors. PLoS One.

[CR3] Andreu-Ballester JC (2022). Changes of CD3+CD56+ gammadelta T cell number and apoptosis during hospital admission are related to mortality in septic patients. Clin Immunol.

[CR4] Basu A (2021). Differentiation and Regulation of T(H) Cells: A balancing act for cancer immunotherapy. Front Immunol.

[CR5] Bellini I et al (2022) Inflammatory response in Caco-2 cells stimulated with Anisakis messengers of pathogenicity. Pathogens 11(10). 10.3390/pathogens11101214 10.3390/pathogens11101214 PMC9611079 36297271

[CR6] Compton CC (2000). Prognostic factors in colorectal cancer. College of American Pathologists Consensus Statement 1999. Arch Pathol Lab Med.

[CR7] Corcuera MT (2018). Exploring tumourigenic potential of the parasite Anisakis: a pilot study. Parasitol Res.

[CR8] Daschner A, Cuellar C, Sanchez-Pastor S, Pascual CY, Martin-Esteban M (2002). Gastro-allergic anisakiasis as a consequence of simultaneous primary and secondary immune response. Parasite Immunol.

[CR9] de Sousa-Pereira P, Woof JM (2019) IgA: Structure, function, and developability. Antibodies (Basel) 8(4). 10.3390/antib8040057 10.3390/antib8040057 PMC6963396 31817406

[CR10] Fleming M, Ravula S, Tatishchev SF, Wang HL (2012). Colorectal carcinoma: pathologic aspects. J Gastrointest Oncol.

[CR11] Fu C (2019). Activation of the IL-4/STAT6 signaling pathway promotes lung cancer progression by increasing M2 myeloid cells. Front Immunol.

[CR12] Garcia-Palacios L, Gonzalez ML, Esteban MI, Mirabent E, Perteguer MJ, Cuellar C (1996). Enzyme-linked immunosorbent assay, immunoblot analysis and RAST fluoroimmunoassay analysis of serum responses against crude larval antigens of Anisakis simplex in a Spanish random population. J Helminthol.

[CR13] Global Burden of Disease Cancer C (2022). Cancer incidence, mortality, years of life lost, years lived with disability, and disability-adjusted life years for 29 cancer groups from 2010 to 2019: a systematic analysis for the Global Burden of Disease Study 2019. JAMA Oncol.

[CR14] Gutierrez R, Cuellar C (2002). Immunoglobulins anti-Anisakis simplex in patients with gastrointestinal diseases. J Helminthol.

[CR15] Hamid HKS (2019). Schistosoma japonicum-associated colorectal cancer: a review. Am J Trop Med Hyg.

[CR16] Hamilton SR, Bosman FT, Boffetta P, Ilyas M, Morreau H, Nakamura SI, Quirke P, Riboli E, Sobin LH, Bosman FTCF, Hruban RH, Theise ND (2010). Carcinoma of the colon and rectum. WHO classification of tumours of the digestive system.

[CR17] Hatta MNA, Mohamad Hanif EA, Chin SF, Neoh HM (2021) Pathogens and carcinogenesis: a review. Biology (Basel) 10(6). 10.3390/biology10060533 10.3390/biology10060533 PMC8232153 34203649

[CR18] Jain S, Rana M (2023). From the discovery of helminths to the discovery of their carcinogenic potential. Parasitol Res.

[CR19] Jain S, Sengupta M, Jain P (2019). Non-viral parasites associated with carcinogenesis. Cancer Invest.

[CR20] Ma R, Yuan D, Guo Y, Yan R, Li K (2020). Immune effects of gammadelta T cells in colorectal cancer: a review. Front Immunol.

[CR21] Maier E, Duschl A, Horejs-Hoeck J (2012). STAT6-dependent and -independent mechanisms in Th2 polarization. Eur J Immunol.

[CR22] Messina CM (2016). Anisakis pegreffii (Nematoda: Anisakidae) products modulate oxidative stress and apoptosis-related biomarkers in human cell lines. Parasit Vectors.

[CR23] Morozinska-Gogol J (2019). Anisakis spp. as etiological agent of zoonotic disease and allergy in European region—an overview. Ann Parasitol.

[CR24] Nappo G (2017). The immunosuppressive cytokine interleukin-4 increases the clonogenic potential of prostate stem-like cells by activation of STAT6 signalling. Oncogenesis.

[CR25] Oines M, Helsingen LM, Bretthauer M, Emilsson L (2017). Epidemiology and risk factors of colorectal polyps. Best Pract Res Clin Gastroenterol.

[CR26] Petithory JC, Paugam B, Buyet-Rousset P, Paugam A (1990). Anisakis simplex, a co-factor of gastric cancer?. Lancet.

[CR27] Poggi A, Zocchi MR (2014). gammadelta T lymphocytes as a first line of immune defense: old and new ways of antigen recognition and implications for cancer immunotherapy. Front Immunol.

[CR28] Redondo F, Hurtado-Marcos C, Izquierdo F, Cuéllar C, Fenoy S, Sáez Y, Magnet Á, Galindo-Regal L, Uribe N, López-Bañeres M, Jiménez AI, Llombart-Cussac A, Del Águila C, Andreu-Ballester JC (2022) Latent Microsporidia Infection Prevalence as a Risk Factor in Colon Cancer Patients. Cancers (Basel). 14(21):5342. https://doi.org/10.3390/cancers14215342 10.3390/cancers14215342 PMC9658866 36358760

[CR29] Sawant M et al (2020) Cryptosporidium and colon cancer: cause or consequence? Microorganisms 8(11). 10.3390/microorganisms8111665 10.3390/microorganisms8111665 PMC7692234 33121099

[CR30] Shang FM, Liu HL (2018). Fusobacterium nucleatum and colorectal cancer: a review. World J Gastrointest Oncol.

[CR31] Silva-Santos B, Mensurado S, Coffelt SB (2019). gammadelta T cells: pleiotropic immune effectors with therapeutic potential in cancer. Nat Rev Cancer.

[CR32] Speciale A (2017). Exposure to Anisakis extracts can induce inflammation on in vitro cultured human colonic cells. Parasitol Res.

[CR33] Sullivan BA, Noujaim M, Roper J (2022). Cause, epidemiology, and histology of polyps and pathways to colorectal cancer. Gastrointest Endosc Clin N Am.

[CR34] Sung H (2021). Global Cancer Statistics 2020: GLOBOCAN estimates of incidence and mortality worldwide for 36 cancers in 185 countries. CA Cancer J Clin.

